# Metagenomic Analysis of Koumiss in Kazakhstan

**DOI:** 10.5195/cajgh.2014.163

**Published:** 2014-12-12

**Authors:** Samat Kozhakhmetov, Indira Tynybayeva, Dinara Baikhanova, Saule Saduakhasova, Gulnar Shakhabayeva, Almagul Kushugulova, Talgat Nurgozhin, Zhaxybay Zhumadilov

**Affiliations:** 1 Center for Life Sciences, Nazarbayev University, Astana, Kazakhstan; 2 Eurasian National University, Astana, Kazakhstan

**Keywords:** fermentation, lactobacteria, DNA sequencing, Kazakhstan

## Abstract

**Introduction:**

Koumiss is a low-alcohol product made from fermented mare’s milk, which is popular in Kazakhstan, Russia, and other countries of Central Asia, China, and Mongolia. Natural mare’s milk is fermented in symbiosis of two types of microorganisms (lactobacteria and yeast). Koumiss’s microbial composition varies depending on the geographical, climatic, and cultural conditions. Based on a phenotypic characteristic from samples, Wu, R. and colleagues identified the following bacteria isolated in inner Mongolia, an autonomous region of China: L.casei, L.helveticus, L.plantarum, L.coryniformis subsp. coryniformis, L.paracasei, L.kefiranofaciens, L.curvatus, L.fermentum, and W.kandleri. Studies of the yeast composition in koumiss also showed significant variations. Thus, there were Saccharomyces unisporus related 48.3% of isolates, to Kluyveromyces marxianus (27.6%), Pichia membranaefaciens (15.0%), and Saccharomyces cerevisiae (9.2%) from 87 isolated yeast cultures. The purpose of this study was to examine the bacterial composition in koumiss.

**Methods:**

To extract DNA, 1.8 ml of fermented milk was centrifuged to generate a pellet, which was suspended in 450 μl of lysis buffer P1 from the Powerfood Microbial DNA Isolation kit (MoBio Laboratories Inc, USA). Amplification of the microflora was used to determine the composition of a fragment of the gene 16S rRNA and ITS1. Plasmid library with target insertion was obtained on the basis of height copy plasmid vectors producing high pGem-T. The definition of direct nucleotide sequencing was performed by the method of Sanger using a set of “BigDye Terminanor v 3.1 Cycle sequencing Kit with automatic genetic analyzer ABI 3730xl (Applied Biosystems, USA). Informax Vector NTI Suite 9, Sequence Scanner v 1.0 software package used for the analysis.

**Results:**

Our studies showed that in the most samples of koumiss isolated from Akmola region (Central Kazakhstan) prevailed the following bacteria species: Lactobacillus diolivorans, Lactobacillus acidophilus, L. casei, L. curvatus yeast genus Torula (62.4%) and Saccharomyces cerevisiae (37.6%).

**Conclusion:**

Thus, the first metagenomic research of koumiss, which was conducted in Kazakhstan, showed significant variations in microbial composition.

